# Computer Vision Syndrome During SARS-CoV-2 Outbreak in University Students: A Comparison Between Online Courses and Classroom Lectures

**DOI:** 10.3389/fpubh.2021.696036

**Published:** 2021-07-08

**Authors:** Lixiang Wang, Xin Wei, Yingping Deng

**Affiliations:** ^1^Department of Ophthalmology, West China Hospital, Sichuan University, Chengdu, China; ^2^Department of Ophthalmology, ShangjinNanfu Hospital, Chengdu, China

**Keywords:** computer vision syndrome, lockdown policy, SARS-CoV-2, online courses, university students

## Abstract

**Purpose:** To compare the prevalence of computer vision syndrome in university students of different teaching modes during the SARS-CoV-2 outbreak period.

**Methods:** A cross-sectional survey study using the validated Computer Vision Syndrome Questionnaire in Chinese medical students of Sichuan University who took classroom lectures and the same-grade foreign students from a Bachelor of Medicine and Bachelor of Surgery (MBBS) program who took online lectures with similar schedules.

**Results:** A total of 137 responses from 63 Chinese students and 74 MBBS students were obtained. The highest frequency of digital screen time was 7-9 h (43.24%, 32/74) for MBBS students and 2-4 h (46.03%, 29/63) for Chinese students. The prevalence of computer vision syndrome among Chinese students and MBBS students were 50.79% and 74.32%, respectively (*P* = 0.004). The average numbers of reported symptoms were 5.00 ± 2.17 in Chinese students and 5.91 ± 1.90 in MBBS students (*P* = 0.01). The three most highly reported symptoms were “heavy eyelids” (53.97%), “dryness” (50.79%), and “feeling of a foreign body” (46.03%) in Chinese students and “dryness” (72.97%), “feeling of a foreign body” (62.16%), and “heavy eyelids” (58.11%) in MBBS students. The sum grades of computer vision syndrome had a moderate positive correlation with screen time (Spearman's correlation coefficient = 0.386, *P* < 0.001). The grades of symptoms of “feeling of a foreign body,” “heavy eyelids,” and “dryness” showed a weak positive correlation with screen time (Spearman's correlation coefficients were 0.220, 0.205, and 0.230, respectively).

**Conclusion:** Online study may contribute to the prevalence of computer vision syndrome among university students.

## Introduction

The ongoing pandemic of SARS-CoV-2 infection has caused tremendous stress in the health care system and altered the daily lifestyle of ordinary people. Episodes of lockdown and social distancing have cut face-to-face connections and interrupted transportation, education, working, and traveling activities. During the lockdown period, people have relied on digital devices for information and entertainment, leading to a higher risk to develop computer vision syndrome, which is often presented with eye stress, dryness, eye pain, and blurred vision (1). Computer vision syndrome is becoming more frequent in modern times due to the widespread use of portable digital terminals and lifestyles that dependent on smartphones (2). There is an increasing concern that the lockdown policy due to the SARS-CoV-2 outbreak may further increase the prevalence of computer vision syndrome in students, because they take extensive online courses and have reduced outdoor activities (3, 4).

In China, the social distancing policy has been gradually lifted since May 2020 and university students have returned to normal school life. However, for foreign students in China, due to the still ongoing traveling restrictions, they are still taking online lectures and tend to spend longer time on digital terminals compared with Chinese students. Thus, we compared the digital screen time and prevalence of computer vision syndrome among Chinese and foreign students who had similar lecture schedules in Sichuan University to assess the impact of school lockdown on the ocular health of students.

## Materials and Methods

A cross-sectional, observational, web-based survey was conducted among a specific group of medical students in Sichuan University in March 2021. The participants included 3rd-grade Chinese students from an 8-year medical program and same-grade foreign students from a Bachelor of Medicine & Bachelor of Surgery (MBBS) program. These students were mainly taking medical courses during the spring semester of 2021. According to the school's academic affairs office, the Chinese and MBBS students took, respectively, 32 and 31 h of lectures per week, corresponding to an average lecture time of 6.4 and 6.2 h per day, respectively. Due to the lockdown policy of COVID-19, foreign students took all lectures online while Chinese students have returned to the university and took lectures in classrooms. Assuming an 80% predictive prevalence (5, 6), a 95% confidence, a 10% margin of error and a 75% response rate, we calculated the sampling size of 82 to be representative of the total population.

The survey was generated by a researcher on an online survey platform (www.wjx.cn) and distributed through class Wechat groups to the students. The survey was composed of three basic parts: anonymous demographic information (gender, nationality, age, and past medical history of ocular diseases), digital terminal use and computer vision syndrome. Computer vision syndrome was evaluated by the Computer Vision Syndrome-Questionnaire (CVS-Q), which included 16 items and has been validated to be a reliable tool for assessing the ocular health of digital terminal users (7). The questionnaire could be independently completed by participants. The English version was downloaded from the original publication and used for foreign students, and the Chinese version was translated by a researcher and used for Chinese students (7). For the screen time, students were asked to evaluate the average time they spent on all digital terminals (computer, phone, television, etc.) per day in the past week after the start of the new semester. The screen time for all activities, including working, studying, and entertainment were included. All intended students were eligible to participate. Exclusion criteria included a history of ocular surgery or active ocular diseases. All participants were informed about the purpose of the study and were free to participate. Digital informed consent was signed online and obtained from all responders. The study was approved by the Ethics Committee of West China Hospital of Sichuan University and was performed in accordance with the declaration of Helsinki.

Data were extracted from the online survey platform and entered into Microsoft Excel software. In the CVS-Q questionnaire, the frequency and intensity of 16 related symptoms were recorded. The frequency score (F) was marked as 0 (never), 1 (sporadic episodes or once a week) or 2 (2 or 3 times a week or almost every day) and the intensity score (I) was marked as 1 (mild to moderate) or 2 (severe). The multiplications of frequency and intensity scores were converted to the grade of each symptom (grade 0: F^*^I = 0, grade 1: F^*^I = 1 or 2, grade 2: F^*^I = 4) according to the instruction of CVS-Q. A participant was considered to have computer vision syndrome if the sum grade of 16 symptoms was ≥6. Data were analyzed with SPSS software version 23.0 (IBM, USA). Digital screen time was described as the number of hours and percentage and compared using a non-parametric test (Mann-Whitney *U*-test). The mean ages of Chinese students and MBBS students were compared with the Student *t*-test. The rates between the two groups were compared using the Chi-square test. The Spearman's correlation test was used to test the correlation between screen time and symptoms of computer vision syndrome. *P* < 0.05 was considered statistically significant.

## Results

### Demographic Features

The survey was distributed to a total of 171 students (83 Chinese students and 88 MBBS students). A total of 137 useful responses were returned by the participants, totaling an overall response rate of 80.12%. The response rates were 75.90% (63/83) for Chinese students and 84.09% (74/88) for MBBS students. Of the total responders, the mean ages were 19.59 ± 0.84 years old (range 18-21) for Chinese students and 18.95 ± 0.76 years old (range 18-20) for MBBS students (*P* < 0.001) while 33.33% of Chinese and 47.30% of MBBS responders were female (*P* = 0.098). The nationalities of MBBS students included Indian (70.27%), Indonesian (12.16%), Sri Lankan (6.76%), Pakistan (6.76%), and others (4.05%).

### Comparison of Digital Screen Time

**Figure 1:**
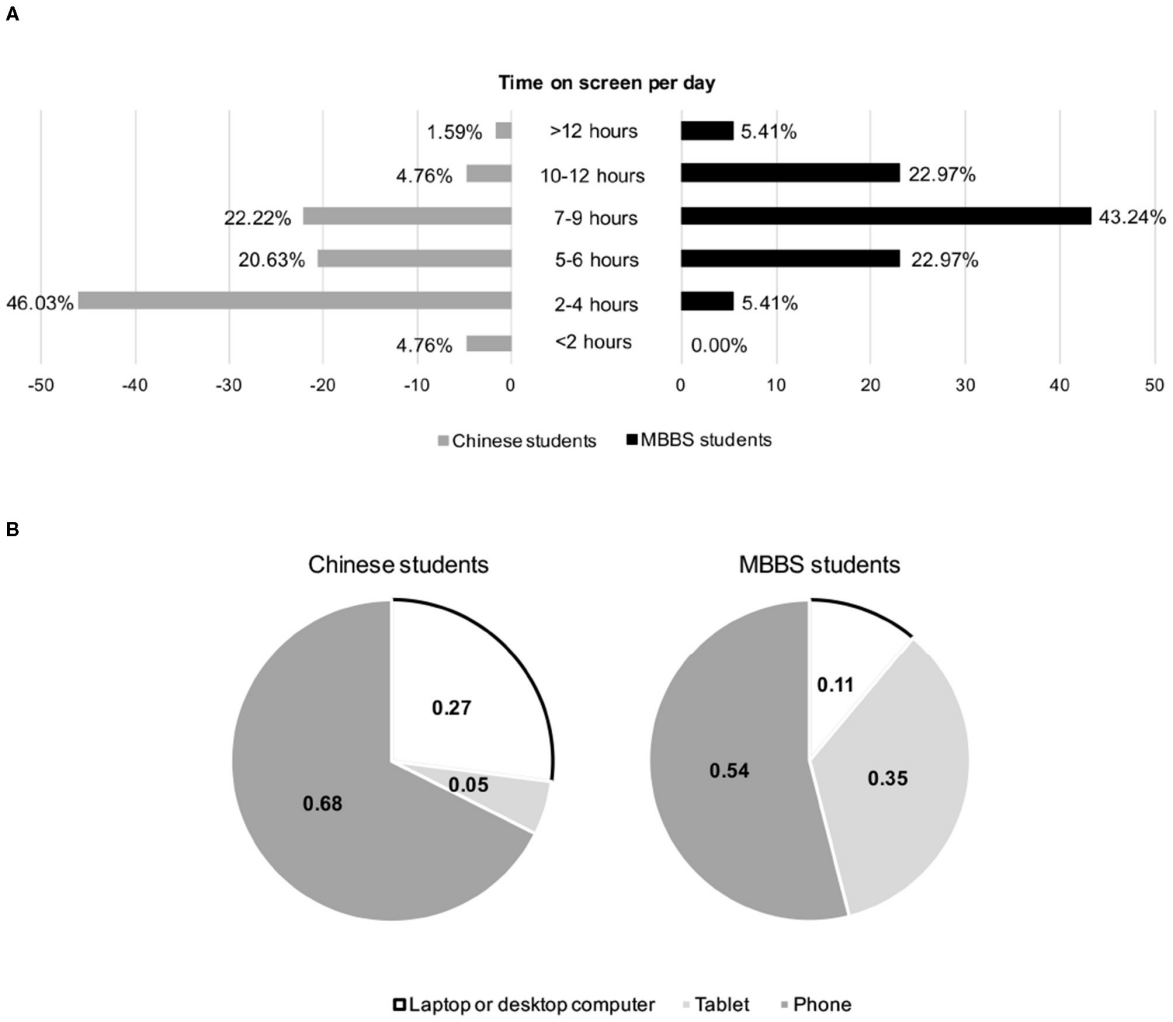
Digital screen time and the use of digital terminals among responders. **(A)** Comparison of average digital screen time per day between Chinese and MBBS students; **(B)** comparison of the most often used digital terminals between Chinese and MBBS students.

The distribution of digital screen time among Chinese and MBBS students is illustrated in Figure 1. For MBBS students, the highest frequency of digital screen time was 7-9 h (43.24%, 32/74) and for Chinese students it was 2-4 h (46.03%, 29/63). The percentage of responders who had a digital screen time of fewer than 5 h per day was 50.79% for Chinese students and only 5.41% for MBBS students. On the other hand, the percentages of heavy users of digital terminals (screen time ≥10 h per day) were 6.35% for Chinese and 28.38% for MBBS students. The overall digital screen time of MBBS students was significantly longer than that of Chinese students (*P* < 0.001). For Chinese students, the digital terminals that were used the most were phones (53.97%), tablets (34.92%), and computers (11.11%) while for MBBS students these were phones (67.57%), computers (27.03%), and tablets (5.41%).

### Comparison of Computer Vision Syndrome

**Figure 2:**
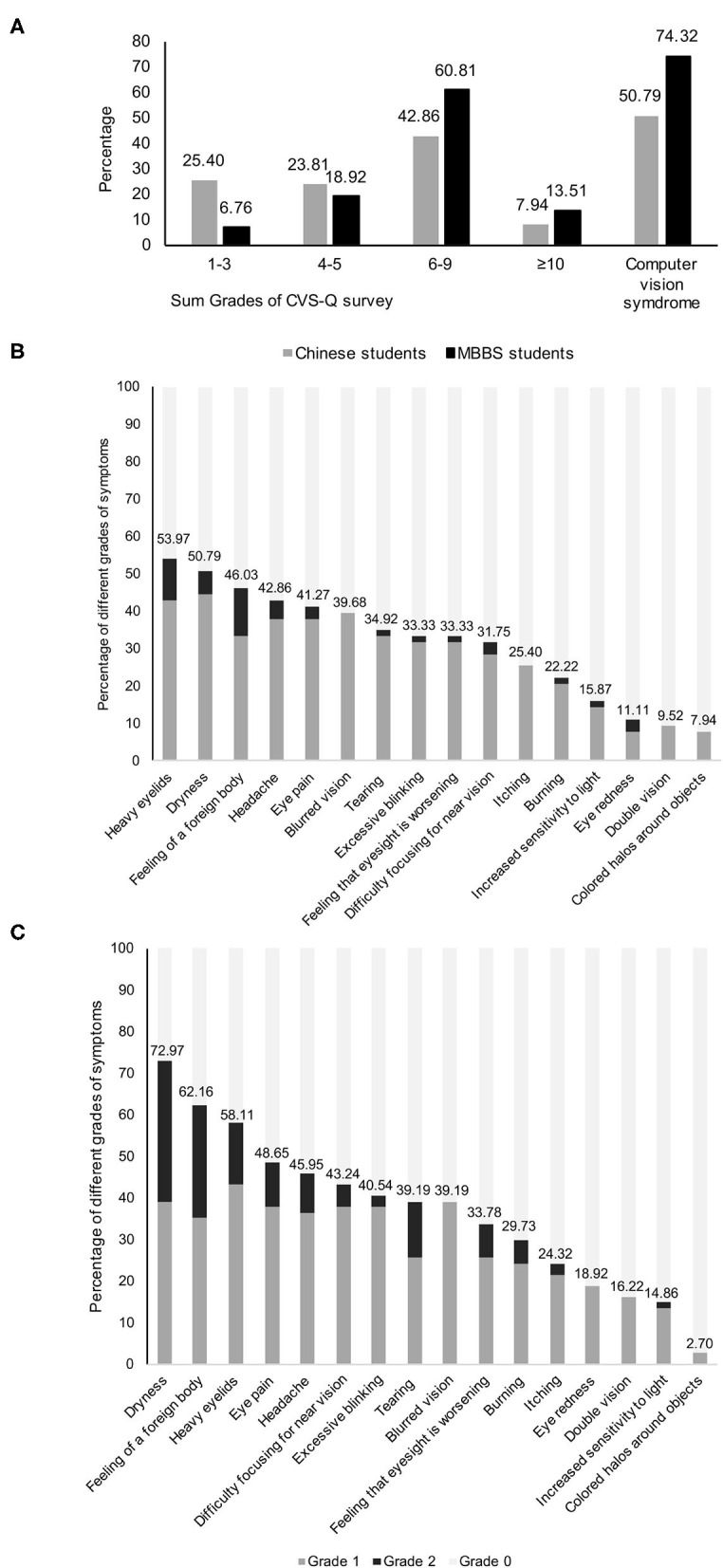
Computer vision syndrome among responders. **(A)** Distribution of sum grades of computer vision syndrome of Chinese and MBBS students; **(B)** grades of each symptom of computer vision syndrome questionnaire in Chinese students; **(C)** grades of each symptom of computer vision syndrome questionnaire in MBBS students.

The grades of each symptom of computer vision syndrome and the sum grades among responders are demonstrated in Figure 2. The prevalence of computer vision syndrome among Chinese and MBBS students was 50.79% and 74.32%, respectively (*P* = 0.004). The percentage of responders with sum grades of computer vision syndrome ≥10 was 7.94% for Chinese and 13.51% for MBBS students (*P* = 0.297). Chinese students reported an average of 5.00 ± 2.17 symptoms, while MBBS students reported an average of 5.91 ± 1.90 symptoms (*P* = 0.010). In Chinese students, the three most commonly reported symptoms were “heavy eyelids” (53.97%), “dryness” (50.79%), and “feeling of a foreign body” (46.03%), while MBBS students reported “dryness” (72.97%), “feeling of a foreign body” (62.16%), and “heavy eyelids” (58.11%). The least reported symptom among both Chinese and MBBS students was “colored halos around objects,” which was reported in 7.94% and 2.70% of responders, respectively.

### Correlation Between Digital Screen Time and Computer Vision Syndrome

The correlation between digital screen time and different symptoms of computer vision syndrome was analyzed by univariate correlations and summarized in Table 1. The digital screen time had a weekly positive correlation with “feeling of a foreign body,” “heavy eyelids,” and “dryness,” and their Spearman's correlation coefficients were 0.220 (*P* = 0.010), 0.205 (*P* = 0.016), and 0.230 (*P* = 0.007), respectively. The correlation of digital screen time with other symptoms was not significant. In addition, the sum grades of computer vision syndrome showed a moderate positive correlation with digital screen time (Spearman's correlation coefficient = 0.386, *P* < 0.001).

**Table 1:** The correlation of digital screen time with grades of symptoms of computer vision syndrome.

**Symptoms of computer vision syndrome**	**Spearman's correlation coefficient**	***P*-value**
Burning	0.126	0.143
Itching	0.056	0.515
Feeling of a foreign body	0.220	0.010
Tearing	0.047	0.584
Excessive blinking	0.069	0.421
Eye redness	0.125	0.144
Eye pain	0.160	0.062
Heavy eyelids	0.205	0.016
Dryness	0.230	0.007
Blurred vision	−0.062	0.475
Double vision	0.060	0.487
Difficulty focusing for near vision	0.159	0.063
Increased sensitivity to light	0.117	0.174
Colored halos around objects	−0.089	0.299
Feeling that eyesight is worsening	−0.020	0.813
Headache	0.100	0.244
Sum grade of computer vision syndrome	0.386	<0.001

## Discussion

In this study, we applied a validated CVS-Q questionnaire to investigate the prevalence of computer vision syndrome among university medical students and studied the impact of school lockdown policy on the ocular health of participants. The overall prevalence of computer vision syndrome was higher (74.32% vs. 50.79%) among MBBS students who took exclusively online courses compared with that of Chinese students who took lectures in classrooms. Due to the online courses, MBBS students spent a much longer time (most frequently 7-9 h per day) on digital screens compared with that of Chinese students (most frequently 2-4 h per day). As the two groups of students spent similar time on common lectures during the semester when the survey was conducted, our study suggested that online study may contribute to the prevalence of computer vision syndrome among students. According to a recent study, students who took online courses had a significantly higher rate of digital eye strain compared with the general public (50.6% vs. 33.2%). The score of the digital eye strain was also found to be the highest among those who had a longer screen time, shorter screen distance and took infrequent breaks (4). In fact, university students have been documented to develop computer vision syndrome potentially due to the study burden and heavy use of digital devices, with a reported high prevalence of 71.6-94.5% (5, 6). To our best knowledge, our study is the first to provide data regarding the impact of online learning during the SARS-CoV-2 outbreak. However, the prevalence of computer vision syndrome in our study was relatively lower than previously reported data. This may in part be due to the different questionnaires used between some studies. Besides, as our study was conducted in the first few weeks of the new semester and students have just returned from a long winter break, so there may be less stress for the students. Our study suggests that some measures, such as promoting regular breaks, setting balanced illumination of screen light and room light, and keeping a suitable distance from the screen should be encouraged to students who take online courses at home (8).

The MBBS and Chinese students have common leading symptoms of computer vision syndrome, including “heavy eyelids,” “dryness,” and “feeling of a foreign body,” but the prevalence of these symptoms was higher in MBBS students. In one previous analysis that included health science students in Saudi Arabia, the three leading symptoms of computer vision syndrome were “headache,” “feeling of an affected eyesight,” and “itchy eye” (9). In another study among Spanish university students, the three symptoms with the highest frequency were “headache,” “itching,” and “heavy eyelids” (10). On the other hand, the least reported symptoms were similar in these studies and included “double vision” and “colored halos around objects.” The difference of the highly reported symptoms such as “itchy eye” in other reports but not in our study may in part be due to the racial difference of participants and the restricted outdoor activities of university students during the SARS-CoV-2 outbreak period. Eye itching is a predominant symptom of ocular allergy and is higher in European countries compared with less developed regions such as south and east Asia (11). Besides, our survey was conducted in early March and students tended to stay indoors more often due to restriction policies. Thus, there was a lower chance of pollen exposure, which may cause ocular allergic symptoms (12). Eye dryness as the 1st and 2nd leading symptom for MBBS and Chines students was, respectively, reported in 72.97% and 50.79% of responders and was relatively more common than other studies (5, 9, 10). This is consistent with findings from the Tear Film and Ocular Surface Society (TFOS) that the Asian race is more likely to develop dry eye disease (13). In our study, the average number of reported symptoms was 5.0 in Chinese students and 5.9 in MBBS students. These numbers were higher than a recent report in the general public during the lockdown period in India, which showed the average numbers of symptoms were 3.5 in females and 2.8 in males (14).

Computer vision syndrome has been documented to be dependent on screen time (15, 16). In consistent with previous findings, our study demonstrated a moderate positive correlation between average screen time and the sum grade of computer vision syndrome. In addition, some specific symptoms, including “feeling of a foreign body,” “heavy eyelids,” and “dryness” were found to be positively associated with screen time. Sustained use of digital terminals, especially without intermittent breaks, has been found to harm ocular health and lead to discomfort by interfering with the tear stability and induce mild inflammation (17). In addition, prolonged starring at digital screens has been found to cause abnormal accommodation and vergence responses, which have been found in nearly 20% of patients with computer vision syndrome (18). In a previous study, participants reading on laptop computers at a distance of 50 cm for 30 min had a mean lag of 0.93D and increased ocular discomfort (19). Besides, there is concern that the exposure to the radiofrequency electromagnetic field emitted from digital terminals may alter the metabolism and structure of ocular surfaces and cause oxidative stress (20, 21). Altogether, these mechanisms may collectively contribute and are responsible for the discomfort experienced in patients with computer vision syndrome.

Our study has some limitations. First, the difference of race, male/female ratios, status of refractive errors, and prior presence of dry eye between Chinese and MBBS students may be confounding factors that may affect the prevalence of computer vision syndrome and susceptibility to certain symptoms such as “dryness” or “itching”. Second, as MBBS students were still restricted from returning to school, we did not conduct further ocular examinations and the evaluation of computer vision syndrome was only based on self-reported symptoms in the questionnaire. Third, the questionnaires in our study were distributed and gathered solely online. This could lead to a selection bias because the students who spent more time on digital devices were more likely to engage in our study and respond. Fourth, the daily digital screen time of students included both the studying activity and that of other purposes. Thus, we could not ascertain the sole impact of online learning on the prevalence of computer vision syndrome.

In conclusion, our study indicated that there is a relationship between online study and prevalence of computer vision syndrome among students during SARS-CoV-2 lockdown. Universities should reinforce the protection of ocular health among students undertaking online courses.

## Data Availability Statement

The original contributions presented in the study are included in the article/Supplementary Material, further inquiries can be directed to the corresponding author/s.

## Ethics Statement

The studies involving human participants were reviewed and approved by Ethics Committee on Biomedical Research, West China Hospital of Sichuan Univeristy. The patients/participants provided their digital informed consent to participate in this study.

## Author Contributions

LW conducted the literature search, distributed and collected questionnaires, and conducted data analysis. XW and YD provided guidance and support for distributing questionnaires and revised the final manuscript. All authors discussed the idea of the study and designed the study protocol.

## Conflict of Interest

The authors declare that the research was conducted in the absence of any commercial or financial relationships that could be construed as a potential conflict of interest.
